# Cetuximab plus oxaliplatin/leucovorin/5-fluorouracil in first-line metastatic gastric cancer: a phase II study of the Arbeitsgemeinschaft Internistische Onkologie (AIO)

**DOI:** 10.1038/sj.bjc.6605521

**Published:** 2010-01-12

**Authors:** F Lordick, B Luber, S Lorenzen, S Hegewisch-Becker, G Folprecht, E Wöll, T Decker, E Endlicher, N Röthling, T Schuster, G Keller, F Fend, C Peschel

**Affiliations:** 1 Klinikum rechts der Isar, 3rd Medical Department, Technische Universität München, Ismaninger Straße 22, 81675 Munich, Germany; 2 Institute of Pathology, Technische Universität München, Ismaninger Straß22, 81675 Munich, Germany; 3 Onkologische Schwerpunktpraxis Eppendorf, Eppendorfer Landstr. 42, 20249 Hamburg, Germany; 4 1st Medical Department, Universitätsklinik Carl Gustav Carus, Fetscherstr.74, 01307 Dresden, Germany; 5 Medical Department, Klinik St. Vinzenz, Sanatoriumstrasse, 6511 Zams, Austria; 6 Onkologische Schwerpunktpraxis, Wilhelm-Hauff-Str. 41, 88214 Ravensburg, Germany; 7 1st Medical Department, Klinikum der Universität, Franz-Josef-Strauss-Allee 11, 93053 Regensburg, Germany; 8 Munich Centre for Clinical Studies, Ismaninger Straße 22, 81675 Munich, Germany; 9 Institute of Medical Statistics and Epidemiology, Technische Universität München, Ismaninger Straße 22, 81675 Munich, Germany; 10 Institute of Pathology, Eberhard-Karls-Universität, Liebermeisterstraße 8, 72076 Tübingen, Germany

**Keywords:** antibody, chemotherapy, cetuximab, EGFR, gastric cancer, *KRAS* mutation

## Abstract

**Background:**

Cetuximab enhances the efficacy of chemotherapy in several cancer types. This trial assessed the activity of cetuximab and chemotherapy in advanced gastric cancer.

**Methods:**

Patients with previously untreated, metastatic, gastric cancer received cetuximab 400 mg m^−2^ at first infusion followed by weekly infusions of 250 mg m^−2^ combined with FUFOX (oxaliplatin 50 mg m^−2^, 5-FU 2000 mg m^−2^, and DL-folinic acid 200 mg m^−2^ d1, 8, 15 and 22 qd36). The primary endpoint was tumour response.

**Results:**

Overall, 52 patients were enrolled. The most common grade 3/4 toxicities were diarrhoea (33%), and skin toxicity (24%). Efficacy was evaluable in 46 patients who showed a response rate of 65% (CI 95%: 50–79%) including four complete responses. Time to progression (TTP) was 7.6 months (CI 95%: 5.0–10.1 months) and overall survival (OS) was 9.5 months (CI 95%: 7.9–11.1 months). Epidermal growth factor receptor (EGFR) was detectable in 60% of tumours but showed no correlation with treatment outcome. A *KRAS* mutation was found in only 1 of 32 (3%) tumour samples analysed.

**Conclusion:**

Cetuximab plus FUFOX showed an interesting high response rate in metastatic gastric cancer. Cetuximab plus platinum–fluoropyrimidine chemotherapy is at present being investigated in a phase III randomised controlled trial.

Stomach cancer is the fourth most common cancer worldwide, with 603 003 new cases in men and 330 290 new cases in women in 2002 (Kamangar *et al*, 2006). Although current fluorouracil- and platinum-based combination chemotherapy regimens confer a survival benefit to patients with advanced gastric cancer when compared with the best supportive care (Wagner *et al*, 2006), the outcomes remain suboptimal. Oxaliplatin has been shown to equal the efficacy of cisplatin in this clinical setting (Al-Batran *et al*, 2008; Cunningham *et al*, 2008). In a previous trial our study group showed that weekly oxaliplatin plus 5-fluorouracil/folinic acid (FUFOX) has a high activity and an acceptable toxicity profile when used in advanced gastric cancer (Lordick *et al*, 2005). This led to a broad acceptance of the FUFOX regimen within the Arbeitsgemeinschaft Internistische Onkologie (AIO) study network and investigators felt that FUFOX can serve as a chemotherapy backbone for combination studies with biologically targeted drugs.

Cetuximab is a monoclonal IgG1 antibody directed against the epidermal growth factor receptor (EGFR) (Martinelli *et al*, 2009). Cetuximab binds to the extracellular domain of EGFR in its inactive configuration and competes for receptor binding by occluding the ligand-binding region. This antibody–receptor interaction prevents receptor dimerisation and thereby blocks ligand-induced EGFR tyrosine kinase activation. Cetuximab also induces EGFR internalisation, downregulation, and degradation. Antibody-dependent cytotoxicity may also contribute significantly to the anticancer activity of cetuximab. By provoking an immune system mediated antitumour response, cetuximab inhibits cancer-cell proliferation (G1 phase arrest), angiogenic growth factor production (VEGF) and tumour-induced angiogenesis, and cancer cell invasion. In preclinical and clinical tumour models cetuximab potentiates the antitumour activity of cytotoxic drugs and radiotherapy (Ciardiello and Tortora, 2008).

In pivotal phase II and phase III studies, cetuximab has shown activity in chemotherapy refractory and chemo-naïve-advanced colorectal cancer given in combination with irinotecan- or oxaliplatin-based chemotherapy (Cunningham *et al*, 2004; Bokemeyer *et al*, 2009; Van Cutsem *et al*, 2009), which is in combination with platinum-based chemotherapy in both advanced non-small-cell lung cancer and squamous cell cancer of the head and neck (Vermorken *et al*, 2008) and in combination with radiotherapy in localised head and neck cancer (Bonner *et al*, 2006).

Epidermal growth factor receptor has been found to be overexpressed in gastric cancer (Gamboa-Dominguez *et al*, 2004; Dragovich *et al*, 2006). Although colorectal tumours with an activating mutation of the *Kirsten(K)-ras* gene are not sensitive to EGFR antibodies (Amado *et al*, 2008; Karapetis *et al*, 2008), the incidence of *KRAS* mutations in gastric cancer appears to be low (Kim *et al*, 2003; Lee *et al*, 2003; Zhao *et al*, 2004).

We carried out a phase II trial to investigate the activity and safety of cetuximab plus weekly FUFOX in patients with metastatic gastric cancer.

## Patients and Methods

This multicentre study was conducted at seven institutions in Germany and Austria. It was organised within the study network of the Arbeitsgemeinschaft Internistische Onkologie (AIO) and was coordinated by the Munich centre for clinical studies. The protocol was approved by the ethics committee for human research at the Technische Universität München, Munich, and conformed to the principles of the Declaration of Helsinki and its subsequent amendments. The study has been assigned the European Clinical Trials Database number 2004-004024-12.

### Patient characteristics

Eligibility criteria included the following: histologically confirmed metastatic or locally advanced irresectable adenocarcinoma of the stomach or oesophago-gastric junction; age 18 years or older; Eastern Cooperative Oncology Group (ECOG) performance status ⩽2; ⩾1 unidimensionally measurable lesion ⩾1 cm in diameter detected by computed tomography (CT) scan or magnetic resonance imaging (MRI); cardiac ejection fraction within normal limits; absolute neutrophil count ⩾2000 *μ*l^−1^; thrombocyte count ⩾100 000 *μ*l^−1^; total bilirubin ⩽1.5 × upper limit of normal (ULN) and transaminases ⩽2.5 × ULN; creatinine clearance >70 ml min^−1^; no previous malignancy and no chemotherapy except in the adjuvant or neoadjuvant setting >6 months before study entry. All patients gave written informed consent.

### Treatment

Cetuximab was administered at an initial dose of 400 mg m^−2^ on day 1 over 120 min, followed by weekly doses of 250 mg m^−2^ over 60 min. Oxaliplatin 50 mg m^−2^ was given i.v. over 120 min followed by folinic acid 200 mg m^−2^ i.v. over 120 min and 5-fluorouracil 2000 mg m^−2^ i.v. over 24 h on days 1, 8, 15, and 22, every 5 weeks (one cycle). Treatment continued until best response, or until there was evidence of disease progression, unacceptable toxicity, death, or withdrawal of patient consent. Toxicity was graded according to the National Cancer Institute Common Toxicity Criteria (NCI-CTC, version 3.0).

### Evaluation

Patients were assessed weekly for potential adverse events and disease-related signs and symptoms. Patients who had ended their treatment but had not experienced disease progression were observed every 12 weeks until progressive disease and every 3 months thereafter. Tumour measurements were undertaken every two cycles (10 weeks) and were carried out according to Response Evaluation Criteria in Solid Tumours (RECIST) criteria (Therasse *et al*, 2000). Best objective responses (primary study endpoint) and confirmed responses were reported.

### EGFR expression analysis

Before randomisation, EGFR expression was evaluated locally from tumour specimens using a standardised immunohistochemistry assay (EGFR pharmDx Dako, Glostrup, Denmark). Positive staining was defined as any membrane staining above background level (defined as the level noted in a negative control sample) in ⩾1% cancer cells of any intensity: with 1+ equating to faint or barely perceptible membrane staining, 2+ indicating weak to moderate staining of the complete cell membrane and 3+ indicating strong staining of the complete cell membrane.

### KRAS mutational analysis

Screening of tumour DNA for *KRAS* mutations in codon 12 or 13 was carried out centrally. Before DNA extraction, tumour tissue (frozen or paraffin-embedded) was manually microdissected to assure at least 60% tumour cell content of the analysed sample. KRAS mutation analysis was carried out by polymerase chain reaction (PCR) amplification of exon 2 and direct sequencing. Primers were modified according to Brink *et al* (2003). DNA sequencing was carried out using the BigDye Terminator v1.1 Cycle Sequencing Kit (Applied Biosystems, Foster City, CA, USA) and separation of the products using an automated sequencing system (3130 Genetic Analyzer, Applied Biosystems).

### Statistical analysis

The primary study endpoint was the proportion of patients who responded to cetuximab–FUFOX. The study was designed as a two-stage trial assuming a response rate of ⩽30% as not being of further interest (null hypothesis) and a response rate of ⩾50% as interesting (alternative hypothesis). The best-observed response was taken as a basis for the determination of primary endpoint and confirmed responses were also reported. The statistical analysis was carried out using SPSS software (version 12.0; SPSS, Chicago, IL, USA). In all, 95% CIs were calculated for all relevant estimates using StatXact (version 5; Cytel, Cambridge, MA, USA). All statistical analyses were carried out at a 5% level of significance.

The secondary study endpoints were median overall survival (OS), time to progression (TTP), and toxicity. Analysis of TTP and OS was carried out using the Kaplan–Meier life-table method. Comparisons between groups of patients were made by log-rank test. Median survival and hazard ratios (HR) calculated by Cox's proportional hazards model were reported, with 95% CIs. TTP and OS were analysed in the intent-to-treat (ITT) population. Time to progression was determined from the day of study assignment to the date of any progression, or last contact. Patients who had not progressed at the time of the final analysis were censored at the date of their last tumour assessment. OS was calculated from the day of assignment to death. Patients alive at the final survival analysis were censored using the last contact date.

## Results

### Patient characteristics

A total of 52 patients were enrolled between April 2005 and March 2006. The primary tumour was located at the oesophago-gastric junction in 25 patients and in other parts of the stomach in the remaining 27 patients. All patients presented with metastatic disease, with the lymph nodes, the liver, the peritoneum, and the lung as the predominant metastatic sites (Table 1).

### Feasibility and safety

In total, 167 cycles of cetuximab–FUFOX were administered, with a median of two cycles (range 0–8) per patient. The median duration of treatment was 10 weeks (range 0–48). In all, 132 of 167 cycles (79%) were given without any delay. At least one dose reduction was carried out in 23 out of 52 patients (44%) because of adverse events, including reductions for one or both cytotoxic drugs (oxaliplatin and/or 5-fluorouracil) in 21 out of 52 patients (40%) and for the antibody cetuximab in 16 out of 52 patients (31%). The main reasons for dose reductions and delays were diarrhoea, haematological side effects, skin toxicity, and fatigue–nausea syndrome.

The study protocol was advised to be continued with treatment until progression. However, 25 patients (48%) discontinued the treatment for other reasons. The main reasons for unplanned therapy discontinuation were as follows: therapy-related adverse events or the deterioration of performance status in 11 patients (21%), request of the patient or withdrawal of consent in 7 patients (13%), achievement of a best clinical response in 3 patients (6%), secondary resection with no further study treatment after surgery in 2 patients (4%), and death without documented progression in 2 patients (4%). Five patients (10%) died within 60 days of study inclusion. The following two deaths were related to treatment: 1 patient died because of a septic shock, which occurred as a result of neutropenic septic diarrhoea; another patient suffered a severe infusion-related hypersensitivity reaction during the first infusion of cetuximab. In rapid succession he developed pneumonia and multi-organ failure and died several days after the first infusion of cetuximab. Two patients died because of early disease progression. One patient rapidly developed symptomatic brain metastases, whereas two patients had rapid progression in extracerebral sites.

### Toxicity

Haematological and non-haematological adverse events are summarised in Table 2. Overall, 34 patients (65% 95% CI, 50–76%) experienced grade 3/4 adverse events. One patient withdrew consent during the first infusion cycle and is therefore excluded from detailed toxicity analyses. The most important grade 3/4 toxicities were: neutropenia 6%, febrile neutropenia 2%, thrombocytopenia 2%, nausea 6%, diarrhoea 33%, sensory neuropathy 4%, hand–foot-syndrome 6%, and skin-reactions 24%.

### Response

In total, 46 patients were assessable for response according to RECIST criteria. A total of 6 patients were not assessable (1 patient died early because of the sequela of an infusion-related reaction, 1 withdrew consent, and 4 patients stopped therapy within the first cycle because of toxicity and were switched to no treatment or other second-line treatment without previous tumour assessment).

The overall response rate (ORR; complete response + partial response) was 65% (95% CI, 50–79%) including 4 complete and 26 partial responses (Table 3). In all, 18 responses have been formally confirmed resulting in a confirmed ORR of 39%. Altogether, 17 out of 22 patients with primary tumours located at the oesophago-gastric junction had an objective response (77%) compared with 13 out of 24 patients (54%) with primary tumours located in the stomach.

### OS and TTP

At a median follow-up of 18 months, 44 out of 52 enrolled patients (85%) presented with progressive disease. At the time of this analysis, 18 patients (35%) are alive.

**Figure 1:**
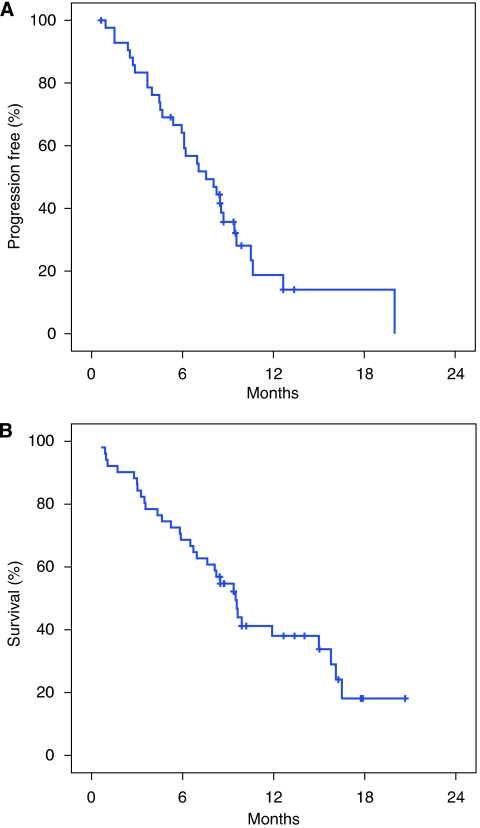
Kaplan–Meier estimates of (**A**) time to progression (TTP) and (**B**) overall survival (OS) among patients with metastatic gastric cancer treated with cetuximab, oxaliplatin, folinic acid, and 5-fluorouracil (FUFOX).

The median TTP was 7.6 months (95% CI: 5.0–10.1 months; Figure 1A). The probability of remaining progression-free at 1 year was 19%.

The median OS was 9.5 months (95% CI: 9.7–11.1 months; Figure 1B). The 1-year survival rate for patients with metastatic disease was 37%.

### EGFR status and *KRAS* mutation analysis

Overall, 42 tumour blocks or slides were available for EGFR analysis and were reviewed centrally at the Institute of Pathology, Technische Universität München. Immunohistochemical EGFR detection was possible in 60% of these samples. No clear association was found between the detection of EGFR and the response rate (Table 4). Of the 32 samples that were analysed for *KRAS* mutation, only one mutation, located in the exon 1 region of the *KRAS* gene, was found.

### Post-progression treatment

Second- or third-line chemotherapy was given to 13 out of 52 patients (25%), consisting mainly of irinotecan (7 patients) and taxanes (3 patients). Three patients received an alternative platinum–fluoropyrimidine-based regimen. Three patients underwent secondary tumour resection in an attempt to achieve a maximum tumour reduction.

## Discussion

Cetuximab, a monoclonal IgG1 antibody has been shown to improve the activity of platinum-based chemotherapy (Vermorken *et al*, 2008; Bokemeyer *et al*, 2009). Epidermal growth factor receptor, the target of cetuximab, is overexpressed in gastric cancer (Gamboa-Dominguez *et al*, 2004; Dragovich *et al*, 2006). Overexpression of EGFR has, in some studies, been associated with a poor prognosis underlining the significant role EGFR may have in human gastric cancer biology. Therefore, it was considered of value to investigate cetuximab in combination with platinum-based combination therapy in patients with advanced gastric cancer. The weekly FUFOX regimen was regarded as an appropriate chemotherapy backbone regimen, as a previous phase II multicentre trial had shown encouraging activity of FUFOX in patients with metastatic gastric cancer (Lordick *et al*, 2005).

The design of this phase II study defined an overall response rate (partial and complete responses) of 30–50% as expected and of 50% or more as being worth further investigation. Response rates of the established chemotherapy doublet regimens used in comparable patient populations have been reported to be below 30% in randomised trials, for example: 20% for cisplatinum and 5-FU in an EORTC study (Vanhoefer *et al*, 2000) and 25% for cisplatin plus 5-fluororacil in the randomised TAX325 study (Van Cutsem *et al*, 2006). Response rates for chemotherapy triplets have been reported to be above 30% but below 50%, for example 46% for epirubicine plus cisplatin and 5-fluorouracil (Waters *et al*, 1999) and 37% for docetaxel plus cisplatin and 5-fluorouracil (Van Cutsem *et al*, 2006). Therefore, we felt that an expected response rate of 30–50% for a chemotherapy doublet plus one biologically targeted agent (‘new triplet’) is an adequate assumption. A response rate of >50% (alternative hypothesis) would be of particular interest for further phase III testing of the new drug.

Treatment with cetuximab plus FUFOX-induced objective tumour responses in 65% of treated patients, which was clearly above the threshold for accepting the alternative hypothesis. It was also 10% higher than the overall response rate that had been observed in the previous FUFOX alone study (Lordick *et al*, 2005). This comparison allows the cautious conclusion that cetuximab may increase the activity of chemotherapy with a platinum compound plus a fluoropyrimidine in advanced gastric cancer.

Our findings are in line with the results obtained by other groups who have studied cetuximab in advanced gastric cancer. Pinto *et al* (2007) were the first to study cetuximab in combination with irinotecan and 5-fluorouracil in this setting and recorded a response rate of 44% with a 95% CI ranging from 28 to 61%, thus indicating the substantial activity of this regimen. The same authors recently published the results of a phase II study combining cetuximab with cisplatin and docetaxel in advanced gastric cancer (Pinto *et al*, 2009). The observed objective response rate of 41% (95% CI, 29–53) was judged to be higher than with cisplatin and docetaxel alone. The TTP in Pinto's first study was particularly interesting as it approached a median of 8 months, which is longer than that achieved with irinotecan and 5-fluorouracil alone (Pozzo *et al*, 2004; Dank *et al*, 2008). These results are supported by the current study in which the median TTP was found to be 7.6 months. A shorter TTP of median 5.5 months, together with a noteworthy response rate of 50%, was reported in the first cetuximab–chemotherapy study published from East Asia (Han *et al*, 2009).

The potentially enhanced anti-tumoural effects of chemotherapy when combined with cetuximab in gastric cancer seen in these clinical trials are not unexpected, given that data from preclinical trials in EGFR-expressing gastric cancer cell lines and tumour xenografts suggested an anti-tumour effect of cetuximab as a result of different intracellular and immunological mechanisms (Hara *et al*, 2008; Shimura *et al*, 2008; Patel *et al*, 2009). Moreover, one recently described mechanism of resistance against cetuximab, that is the activating mutation of the *KRAS* gene, seems to have a very low prevalence in gastric cancer (Kim *et al*, 2003; Lee *et al*, 2003; Zhao *et al*, 2004). This finding has been confirmed in the current study, wherein a *KRAS* mutation was found in only one tumour sample.

This study revealed that cetuximab plus FUFOX is a feasible regimen associated with the generally manageable toxicity. However, the rate and the severity of observed side effects and adverse events were higher than that reported with FUFOX alone. The incidence of diarrhoea (33% grade 3/4), in particular, seemed to be increased with the addition of cetuximab and the different phenotypes of skin toxicities (24% grade 3/4) associated with the use of anti-EGFR-directed drugs must also be taken into consideration. The patients enrolled in this trial presented with a high tumour load. All patients had metastatic disease, no patients with locally advanced tumours were included, and almost 50% had more than two organ systems involved. Compared with other phase II studies like the FUFOX alone study published previously (Lordick *et al*, 2005), patients presented with a poorer performance status and many had been pretreated with adjuvant chemotherapy. The poorer performance status and more advanced disease status of patients included in this study is also reflected by the 60-day mortality rate of 10% that was higher than in previously reported phase II trials of the same group of authors (Lordick *et al*, 2005; Lorenzen *et al*, 2007). This may explain the short duration of treatment in a considerable number of patients. Tragically, one patient died because of the sequelae of a severe infusion-related drug reaction occurring during the first infusion of cetuximab. This appalling event underlines the necessity of prophylactic premedication with corticosteroids and antihistamines in patients undergoing the first infusion with cetuximab and the unconditional need for continuous cardiorespiratory monitoring and direct observation.

New biologically targeted agents are currently being investigated in gastric cancer. The results of this study, together with Pinto *et al's* study published previously, encouraged us to initiate a randomised, multinational, phase III study to investigate the value of cetuximab in combination with platinum–fluoropyrimidine–chemotherapy in stage IV gastric cancer.

## Figures and Tables

**Table 1:** Patient and tumour characteristics at baseline

**Characteristic**	**No of patients (*n*=52)**	**%**
*Age (years)*		
Median	63	
Range	38–80	
		
*Gender*		
Male	39	75
Female	13	25
		
*ECOG performance status*		
0	19	37
1	25	48
2	8	15
		
*Disease status*		
Locally advanced	0	0
Metastatic	52	100
		
*Metastatic sites*		
Lymph node	45	87
Liver	23	46
Lung	9	17
Peritoneum	16	31
Bone	4	8
Other	5	10
		
*Site of the primary tumour*		
Oesophago-gastric junction	25	48
Stomach	27	52
		
*No. of organs involved*		
1	10	19
2	20	39
>2	22	42
		
*Previous therapy*		
Chemotherapy (adjuvant)	12	23
Radiotherapy	3	6
Surgery	26	52

Abbreviation: ECOG=Eastern Cooperative Oncology Group.

**Table 2:** Haematological and non-haematological toxicities (National Cancer Institute Common Toxicity Criteria, Version 3.0)

	**No. of patients (%) (*n*=51)**		
**Toxicity**	**Grade 1–2**	**Grade 3–4**	**Total**
Haematological toxicity			
Anaemia	42 (82)	0 (0)	42 (82)
Neutropenia	23 (45)	3 (6)	26 (51)
Febrile neutropenia	—	3 (5)	3 (5)
Thrombocytopenia	15 (29)	1 (2)	16 (31)
Non-haematological toxicity			
Diarrhoea	26 (51)	17 (33)	43 (84)
Nausea	28 (54)	3 (6)	31 (60)
Emesis	15 (29)	0 (0)	15 (29)
Skin toxicity	33 (65)	12 (24)	47 (89)
Sensory neuropathy	33 (65)	2 (4)	35 (69)
Hand–foot syndrome	16 (31)	3 (6)	19 (37)
Asthenia	30 (59)	5 (10)	35 (69)

**Table 3:** Anti-tumour activity (evaluable patients, *n*=46)

**Response according to RECIST**	**No. of patients**	**%**
Overall response rate	30	65
Complete response	4	9
Partial response	26	57
Stable disease	8	17
Progressive disease	8	17

Abbreviation: RECIST=Response Evaluation in Solid Tumours.

**Table 4:** Clinical outcome according to EGFR immunohistochemistry

	**EGFR detectable**	**EGFR non-detectable**
Overall response rate	54%	76%
Clinical benefit rate (CR, PR, and SD)	79%	82%
Median time to progression	7.0 months	9.4 months
Median overall survival	8.1 months	9.9 months

Abbreviations: CR=complete response; EGFR=epidermal growth factor receptor; PR, partial response; SD=stable disease.
